# The incidence, risk factors and outcomes of impaired cerebral autoregulation in aortic arch surgery: a single-center, retrospective cohort study

**DOI:** 10.1186/s13019-023-02413-z

**Published:** 2023-11-10

**Authors:** Ling Peng, Dan Guo, Yinhui Shi, Jiapei Yang, Wei Wei

**Affiliations:** Department of Anesthesiology, West China Hospital, Sichuan University, 37 Guo Xue Xiang, Chengdu, 610041 China

**Keywords:** Cerebral autoregulation, Cardiopulmonary bypass, Hypothermia circulatory arrest, Near-infrared spectroscopy

## Abstract

**Background:**

Impairment of cerebral autoregulation (CA) has been observed in patients undergoing cardiopulmonary bypass (CPB), but little is known about its risks and associations with outcomes. The cerebral oximetry index (COx), which is a moving linear correlation coefficient between regional cerebral oxygen saturation (rScO_2_) and mean blood pressure (MAP), may reflect CA function. When COx approaches 1, it implies that CA is damaged, whereas the CA is functional when the COx value approaches 0. The objective of this study was to analyze the incidence and risks of impaired CA, based on COx assessment, in patients undergoing total aortic arch replacement under systemic moderate hypothermia and circulatory arrest of the lower body (MHCA). We also evaluated the association between impaired CA and patient outcomes.

**Methods:**

One hundred and fifty-four adult patients who underwent total aortic arch replacement with stented elephant trunk implantation under MHCA at our hospital were retrospectively analyzed. Patients were defined as having new-onset impaired CA if pre-CPB COx < 0.3 and post-CPB COx > 0.3. Pre- and intraoperative factors were tested for independent association with impaired CA. Postoperative outcomes were compared between patients with normal and impaired CA.

**Results:**

In our 154 patients, 46(29.9%) developed new-onset impaired CA after CPB. Multivariable analysis revealed a prolonged low rScO_2_ (rScO_2_ < 55%) independently associated with onset of impaired CA, and receiver operating charactoristic curve showed a cutoff value at 40 min (sensitivity, 89.5%; specificity, 68.0%). Compared with normal CA patients, those with impaired CA showed a significantly higher rates of in-hospital mortality and postoperative complications.

**Conclusions:**

Prolonged low rScO_2_ (rScO_2_ < 55%) during aortic arch surgery was closely related to onset of impaired CA. Impaired CA remained associated with the increased rates of postoperative complications and in-hospital mortality.

**Trial registration:**

ChiCTR1800014545 with registered date 20/01/2018.

## Background

Cerebral autoregulation (CA) ensures a constant supply of oxygenated blood flow to the brain over a wide range of blood pressures [1]. However, when CA is damaged, cerebral blood volume (CBV) may become correlated with blood pressure, leading to cerebral hypo- or hyperperfusion in patients whose blood pressure is uncontrolled. It also predisposes patients with low blood pressure to cerebral ischemia and patients with high blood pressure to hyperemia [1]. CA may become damaged in up to 20–24% of patients undergoing mild hypothermic cardiopulmonary bypass (CPB) [1, 2].

Impaired CA has been linked to neurological dysfunction in patients undergoing hypothermic CPB [1, 3]. Brain ischemic injury with low arterial pressure and increased cerebral embolic load with high arterial pressure are proposed mechanisms of neurological dysfunction in patients with impaired CA [1]. Whether CPB involving hypothermic circulatory arrest (HCA) of lower body and selective cerebral perfusion increase the risk of impaired CA in aortic dissection patients is unclear. On the one hand, Neri et al’s work revealed that HCA combined with retrograde cerebral perfusion might damage CA [4]. And on the other hand, Ono et al’s study indicated that deep HCA could preserve CA better than moderate hypothermic CPB without circulatory arrest [5]. Therefore, the effect of HCA on CA remains unclear and requires further investigation.

Regional cerebral oxygen saturation (rScO_2_) monitoring using near-infrared spectroscopy (NIRS) has been widely applied in cardiac surgeries, carotid endarterectomy, and shoulder surgeries in beach-chair position [6–9]. rScO_2_ takes into account cerebral arterial, capillary, and venous blood, essentially reflecting the balance between cerebral oxygen supply and demand [10]. Particularly for patients who underwent total aortic arch replacement under HCA, rScO_2_ monitoring could help to manage the flow rate of cerebral perfusion [11–13]. In previous research, it has been demonstrated that the change of rScO_2_ was coherent with CBV in patients undergoing CPB or in those with an intracranial injury [14, 15]. And the function of CA can be assessed by measuring a moving linear correlation coefficient between rScO_2_ and mean blood pressure (MAP), which is called the cerebral oximetry index (COx) [14]. If the COx approached 1, it implied that CBV depended on blood pressure and CA was damaged. If the COx value approached 0, it indicated that blood pressure did not correlate with CBV and CA was functional. An average COx > 0.3 was regarded as the threshold of impaired CA [5]. Furthermore, COx analysis has shown high sensitivity (92%) and moderate specificity (63%) for detecting CA impairment [16], and it agrees well with the mean velocity index (Mx) determined by transcranial Doppler (TCD) [14, 17]. The feasibility of using COx to monitor CA during cardiac surgery has been demonstrated for adult and pediatric patients [18, 19].

In this retrospective study, we aimed to identify the incidence and potential risk factors for new-onset impaired CA by COx calculation in patients undergoing total aortic arch replacement involving moderate hypothermic circulatory arrest (MHCA) of lower body and selective cerebral perfusion. We hypothesized that MHCA would have an effect on CA by comparison of COx levels before and after CPB. We also analyzed the associations between impaired CA and short-term outcomes.

## Methods

### Study design and population

We retrospectively reviewed the electronic medical records of adult patients who underwent total aortic arch replacement with stented elephant trunk implantation for acute type A aortic dissection from February 2017 to December 2018. This study was approved by the Ethics Committee of West China Hospital, Sichuan University (protocol number: 2,017,342). Written informed consent was waived because of retrospective and observational study. All procedures performed in studies involving human participants were in accordance with the Helsinki declaration. Furthermore, the study was registered in the chictr.org.cn with registration number: ChiCTR1800014545.

### Perioperative care and anesthesia

Five-lead electrocardiography (ECG), pulse oxygen saturation (SpO_2_), nasopharyngeal and rectal temperature, and invasive blood pressures via the bilateral radial arteries and left dorsal pedis artery were routinely monitored. General anesthesia was induced using midazolam (0.04–0.1 mg/kg), sufentanil (1–2 µg/kg), and rocuronium (0.5–1.2 mg/kg), then maintained using sevoflurane inhalation (1–2%) and intermittent administration of sufentanil and cisatracurium besilate. After tracheal intubation, pressure-controlled mechanical ventilation was achieved and adjusted to keep end-tidal carbon dioxide (EtCO_2_) in the normal range. Transesophageal echocardiographic examination (iE33; Phillips Medical System, Andover, MA, USA) was routinely performed before surgery. Vasoactive agents were administrated necessarily to stabilize hemodynamics as much as possible.

### Surgical procedures

All patients underwent total aortic arch replacement with stented elephant trunk implantation through median sternotomy in supine position. Aortic cannulation, right axillary artery, or femoral artery cannulation was performed for systemic perfusion, and systemic venous return was achieved by vena cava cannulation or trans-femoral venous cannulation. Systemic moderate hypothermia (nasopharyngeal temperature 26–28 °C and rectal temperature 28–30 °C) was reached before the establishing circulatory arrest of the lower body. During the cooling phase before MHCA, the pump flow rate decreased gradually from 2.6 to 2.2 L/min/m^2^. If MAP lower than 50 mmHg, vasoconstrictor, including metaraminol (0.2–0.5 mg) or norepinephrine (5–10 µg), was administrated intermittently; when MAP higher than 80 mmHg, vasodilator, including urapidil (3–5 mg) or perdipine (0.3–0.5 mg) was used. After the establishment of MHCA, selective antegrade cerebral perfusion (ACP) was performed initially via innominate artery cannulation. If left rScO_2_ was 10% lower than right rScO_2_ during right ACP, unilateral ACP was immediately switched to bilateral ACP through both innominate artery and left common carotid artery cannulations to improve cerebral oxygenation as much as possible. The flow rate of ACP was adjusted between 6 and 12 mL/min/kg under the guidance of right radial artery blood pressure or perfusion pressure. The right radial artery pressue was maintained between 40 and 70 mmHg as possible, while the cerebral perfusion pressure was kept between 40 and 50 mmHg. Alpha–stat management was used during the cooling and rewarming phases, while pH-stat was applied during the MHCA and selective ACP phases. All patients were transferred to the intensive care unit (ICU) after surgery for respiratory and circulatory support.

### rScO_2_ monitoring and COx calculation

**Fig. 1:**
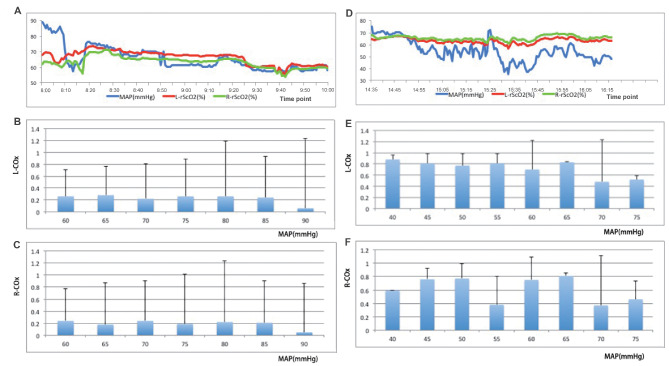
Examples of rScO_2_, MAP and COx recordings in a patient with normal CA before CPB but who became impaired after the CPB. A shows MAP, L-rScO_2_ and R-rScO_2_ recordings before CPB. B shows L-COx values in MAP bins of 5 mmHg before CPB. C shows R-COx values in MAP bins of 5 mmHg before CPB. D shows MAP, L-rScO_2_ and R-rScO_2_ recordings after CPB. E shows L-COx values in MAP bins of 5 mmHg after CPB. F shows R-COx values in MAP bins of 5 mmHg after CPB. rScO_2_, regional cerebral oxygen saturation; MAP, mean arterial blood pressure; COx, cerebral oximetry index; CA, cerebral autoregulation; CPB, cardiopulmonary bypass; L-rScO_2_, regional cerebral oxygen saturation of left frontal cerebral; R-rScO_2_, regional cerebral oxygen saturation of right frontal cerebral; L-COx, cerebral oximetry index of left cerebral; R-COx, cerebral oximetry index of right cerebral

Two self-adhesive transcutaneous oximetry sensors (EGOS-600 A, Suzhou Engine Bio-medical Electronics, Suzhou, China) were placed on the right and left sides of the forehead for bilateral rScO_2_ monitoring. MAPs and rScO_2_ were sampled with an analog-to-digital converter at 60 Hz and then processed with SAM 1.0 software (Senton Netease, Chengdu, China) and the EGOS-600 A system respectively. For COx calculation, the saved MAP and rScO_2_ data were extracted and redisplayed by Visual Studio 2013 software (Microsoft Corporation, WA, USA) on a personal computer (Lenovo XiaoXin Air 13 Pro). Of note, the MAP, measured in the left radial artery, was preferred for COx calculation. A continuous, moving Pearson correlation coefficient between MAP and rScO_2_ was calculated to generate COx [14]. Consecutive, paired, non-overlapping 10-second mean values of MAP and rScO_2_ were calculated over 300-sec interval, yielding 30 data points, which were used to determine the COx for that interval. This operation was equivalent to applying a moving filter with a 10-second time window and resampling at 0.1 Hz to eliminate high-frequency noise at the same time as allowing detection of oscillations and transients that occur below 0.05 Hz. Then, the mean values of COx of all 300-sec intervals for the pre- and post-CPB periods were used respectively to identify impaired CA. A COx near 1 indicates that CBV depends on blood pressure and so CA is damaged; a COx near 0 indicates that CBV does not correlate with blood pressure and therefore CA is functional [14]. New-onset impaired CA was defined as both right and left mean value of COx > 0.3 after CPB and ≤ 0.3 before CPB at all recorded MAPs [5]. Figure 1 shows one patient’s COx data in MAP bins of 5mmHg. The threshold of low rScO_2_ was defined as lower than 55% according to that rScO_2_ below 55% was related to the occurrence of neurological events [20, 21].

### Data collection and definition

Preoperative variables were age, body mass index, sex, ejection fraction (EF), presence of comorbidities (diabetes, hypertension), baseline creatine, baseline hemoglobin, baseline b-type natriuretic peptide, and preoperative medication. Intraoperative variables were type of cerebral perfusion and systemic perfusion, MAP, central venous pressure, operation time, CPB time, cross-clamp time, cerebral perfusion time, red blood cells transfusion, temperatures and blood gas parameters during HCA, and rScO_2_ values.

Postoperative outcomes were major complications including delirium, stroke, acute kidney injury (AKI), cardiac dysfunction, mechanical ventilation > 24 h, respiratory infection, and reoperation. Lengths of stay in the ICU and hospital generally were also recorded. Postoperative delirium was measured with the Confusion Assessment Method (CAM) or CAM-ICU for intubated patients. Stroke is defined as a global or focal neurological lesion, mainly detected by cerebral computed tomography (CT). AKI was diagnosed according to the Kidney Disease Improving Global Outcomes (KDIGO) criteria as a 50% increase from baseline serum creatinine level or a 26.4 mmol/L increase from baseline within 48 h [22]. Cardiac dysfunction was defined as the minimal EF < 50% during postoperative hospitalization. Postoperative respiratory infection was identified as follows: if a patient received antibiotics for suspected respiratory infection and met at least one of the following criteria: new or changed sputum, new or changed lung opacities, fever, leukocyte count > 12,000 × 10^9^ L^− 1^ [23].

### Statistical analysis

Continuous variables were expressed as mean ± standard deviation (SD), and categorical data as frequency in percentage or absolute number. Normality of the continuous data was tested using the Kolmogorov-Smirnov method. Inter-group differences in continuous variables were assessed for significance using Student’s *t*-test, and differences in categorical variables were assessed using χ^2^ or Fisher’s exact test. Preoperative and intraoperative variables were then entered into a univariable logistic regression model to assess for a relationship between each variable and impaired CA. Covariates with an explanatory *P < 0.10* were then manually entered into a multivariable logistic regression model. In cases of intercorrelation, the best single independent variable was chosen. For the predictors of impaired CA, adequate cutoff values were indentified using a receiver operating charactoristics curve. According to the previous study, impaired CA occurred in 20% of patients underwent CPB, and the odds ratio was 2 for PaCO_2_ corresponding to impaired CA [1]. A sample size of 136 patients achieves 90% power at 0.05 significance by logistic regression analysis. Statistical analyses were performed using SPSS version 17.0 (IBM, Chicago, IL, USA), GraphPad Prism 7.0 (GraphPad Software, USA), and PASS 15.0 software. Differences with *P* < 0.05 were considered statistically significant.

## Results

**Fig. 2:**
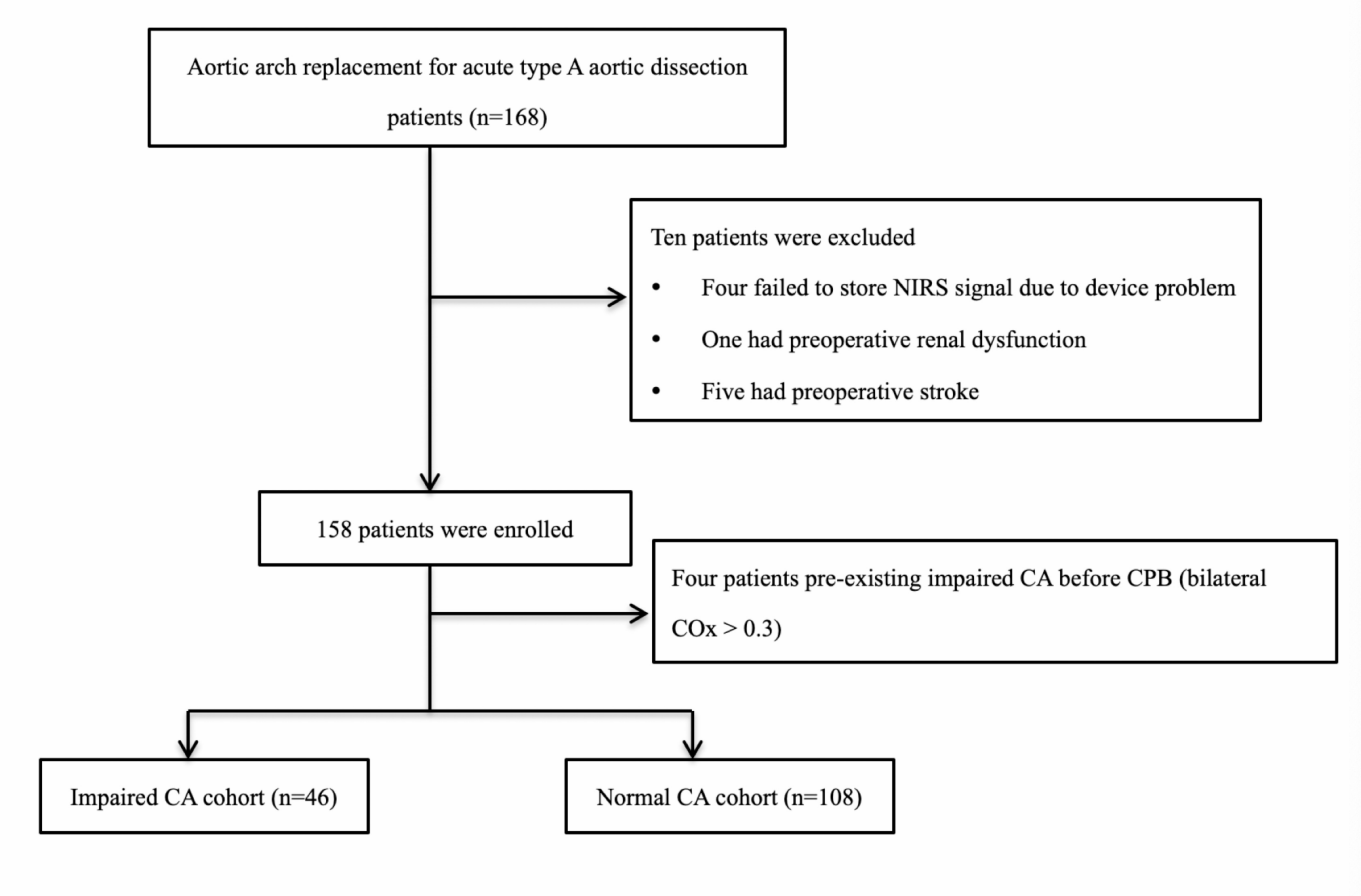
Flow chart of patient selection. CPB, cardiopulmonary bypass; HCA, hypothermic circulatory arrest; CA, cerebral autoregulation; NIRS, near-infrared spectroscopy

One hundred and fifty-four adult patients underwent total aortic arch replacement with MHCA were reviewed. Patients with missing NIRS data (n = 4), preoperative renal dysfunction (n = 1), preoperative stroke (n = 5), and impaired CA prior to CPB (pre-CPB COx > 0.3) (n = 4) were excluded. Finally, 154 cases were enrolled in this study, of which 46 (29.9%) patients presented with new-onset impaired CA after MHCA (Fig. 2).

The preoperative profile and intraoperative data of the two cohort were listed in Table 1. There was no significant difference in the preoperative state between the 2 cohorts. In regards to their intraoperative course, patients who developed new-onset impaired CA had longer antegrade cerebral perfusion time and low rScO_2_ (rScO_2_ < 55%) duration, as well as lower mean value of rScO_2_ than normal CA patients. The ACP pattern, intraoperative MAP, and CVP showed no significant difference between the two cohorts. Moreover, there were no differences in temperature, pH, carbon dioxide partial pressure (PaCO_2_), arterial oxygen partial pressure (PaO_2_), lactate, or hemoglobin between patients with impaired and normal CA during MHCA (Table 2).

**Table 1:** Preoperative and intraoperative characteristics of the patients with impaired or normal cerebral autoregulation

Parameter	Impaired autoregulation (n = 46)	Normal autoregulation (n = 108)	*P* value
Frequency (%)	29.9	70.1	
**Baseline characteristics**			
Age (years)	49.5 ± 11.2	47.2 ± 10.8	0.225
BMI (kg/m^2^)	29.3 ± 4.3	25.0 ± 3.7	0.084
Female, n (%)	6(13.0)	21(19.4)	0.339
Preoperative medication, n (%)			
β-blockers	12 (26.3)	22(20.3)	0.434
ACEI	19 (41.3)	38 (35.1)	0.472
CCB	24 (52.1)	43 (39.8)	0.157
Insulin	7 (15.2)	13 (12.0)	0.668
Creatinine (µmol/L)	117.5 ± 121.1	100.6 ± 63.6	0.263
Hemoglobin (g/L)	119.6 ± 22.8	124.5 ± 25.3	0.263
BNP (pg/mL)	1063.1 ± 1643.0	987.8 ± 1981.6	0.826
Diabetes, n (%)	19 (41.3)	36(33.3)	0.345
Hypertension, n (%)	34 (73.9)	71 (65.7)	0.319
Ejection fraction < 50%, n (%)	4(8.7)	9(8.3)	0.941
Moderate to massive pericardial effusion, n (%)	7(15.2)	13(12.0)	0.556
Emergency surgery, n (%)	22 (47.8)	37 (35.2)	0.113
**Intraoperative factors**			
ACP			
uACP, n (%)	39(84.7)	97(89.8)	0.374
biACP, n (%)	7(15.2)	11(10.2)	0.374
Systemic perfusion			
Trans-femoral artery, n (%)	34(73.9)	73(67.6)	0.531
Trans-aorta, n (%)	12(26.1)	35(32.4)	0.531
MAP (mmHg)			
Pre-CPB	62.3 ± 6.3	62.0 ± 7.6	0.893
During CPB	48.0 ± 5.7	51.1 ± 6.7	0.086
Post-CPB	52.0 ± 3.9	53.9 ± 4.6	0.090
CVP (cmH_2_O)			
Pre-CPB	5.7 ± 3.2	7.2 ± 3.8	0.211
Post-CPB	8.1 ± 3.3	10.2 ± 3.5	0.071
Operation time (min)	476.9 ± 100.8	456.6 ± 85.7	0.206
CPB time (min)	257.9 ± 56.2	253.9 ± 73.3	0.754
Pre-CPB time (min)	147.8 ± 32.8	148.3 ± 29.7	0.958
Post-CPB time (min)	144.1 ± 47.9	127.1 ± 48.0	0.136
Cross-clamp time (min)	187.5 ± 49.1	175.7 ± 50.5	0.228
ACP time (min)	37.7 ± 9.1	33.4 ± 9.2	0.008
RBC infusion, n (%)	6 (31.6)	11 (24.4)	0.604
Left rScO_2_ baseline (%)	61.7 ± 4.5	60.9 ± 3.8	0.562
Right rScO_2_ baseline (%)	59.6 ± 6.2	61.7 ± 4.5	0.167
Left rScO_2_ minimum (%)	55.3 ± 3.7	55.1 ± 5.6	0.900
Right rScO_2_ minimum (%)	55.4 ± 4.6	54.9 ± 5.1	0.254
Left rScO_2_ mean (%)	57.2 ± 3.6	59.9 ± 5.1	0.041
Right rScO_2_ mean (%)	57.6 ± 4.8	60.4 ± 4.3	0.035
Left rScO_2_ < 55% duration (min)	71.5 ± 36.4	33.5 ± 44.2	0.002
Right rScO_2_ < 55% duration (min)	67.6 ± 24.9	33.5 ± 45.3	0.003
Left rScO_2_ < 50% duration (min)	4.6 ± 13.0	7.2 ± 20.8	0.627
Right rScO_2_ < 50% duration (min)	14.5 ± 39.1	11.1 ± 33.8	0.773

Values are n (%) or mean ± SD, unless otherwise noted

Abbreviations: BMI, body mass index; ACEI, angiotensin-converting enzyme inhibitor; CCB, calcium-channel blocker; BNP, b-type natriuretic peptide; ACP, antegrade cerebral perfusion; uACP, unilateral antegrade cerebral perfusion; biACP, bilateral antegrade cerebral perfusion; MAP, mean arterial blood pressure; CVP, central venous pressure; CPB, cardiopulmonary bypass; RBC, red blood cell

*P* < 0.05

**Table 2:** Temperatures and blood gas analysis of patients averaged over hypothermic circulatory arrest, stratified by impaired or normal cerebral autoregulation

Parameters	Impaired autoregulation (n = 46)	Normal autoregulation (n = 108)	*P* value
Nasopharyngeal T_mean_(℃)	26.5 ± 1.7	26.8 ± 2.2	0.522
Nasopharyngeal T_min_(℃)	26.3 ± 1.7	26.5 ± 2.3	0.732
Rectal T_mean_(℃)	28.1 ± 2.1	28.5 ± 2.0	0.442
Rectal T_min_(℃)	27.9 ± 2.0	28.4 ± 1.9	0.407
pH	7.2 ± 0.1	7.2 ± 0.1	0.755
PaCO_2_ (mmHg) PaO_2_ (mmHg)	62.2 ± 13.7 191.3 ± 85.3	59.6 ± 13.0 221.2 ± 93.6	0.519 0.251
Lactate (mmol/L)	4.8 ± 2.0	5.4 ± 2.7	0.447
BE	-3.6 ± 2.3	-5.0 ± 2.6	0.056
Hemoglobin (g/L)	78.0 ± 10.2	82.2 ± 11.0	0.180
Glucose (mmol/L)	9.8 ± 2.7	11.0 ± 4.1	0.200

Values are mean ± SD, unless otherwise noted

Abbreviations: T, temperature; PaCO_2_, partial pressure of carbon dioxide; PaO_2_, partial pressure of oxygen; BE, base excess

*P* < 0.05

The variables listed in Table 1 were tested for univariable association with impaired CA (Table 3). On univariable analysis, only those varibles including bilateral rScO_2_ mean values and durations of bilateral rScO_2_ < 55% were significant. The risk variables with an explanatory *P < 0.10* at the univariable step were tested with a multivariable analysis. The variables identified in the univariable analysis were tested for intercorrelation. There was a significant correlation between left rScO_2_ mean value and right rScO_2_ mean value and between left rScO_2_ < 55% duration and right rScO_2_ < 55% duration. We therefore included left rScO_2_ mean value and left rScO_2_ < 55% duration in the multivariable models because right side selective cerebral perfusion was mostly performed. After correction for other explanatory facotrs, left rScO_2_ < 55% duration independently associated with the occurrence of impaired CA.

**Table 3:** Univariable and Multivariable Analysis for predictors of impaired cerebral autoregulation

Variables	Odds Ratio	95% Confidence Interval	P-value
**Univariate Analysis**			
Age (y)	1.014	0.977–1.053	0.464
BMI (kg/m^2^)	1.046	0.931–1.176	0.449
Female	1.922	0.636–5.805	0.247
Hemoglobin (g/L)	0.989	0.971–1.006	0.207
Diabetes (Absent, present)	0.791	0.322–1.884	0.596
Hypertension (Absent, present)	0.853	0.347–2.101	0.730
CPB time (min)	1.001	0.995–1.006	0.752
Cross-clamp time (min)	1.005	0.997–1.013	0.228
ACP time (min)	1.038	0.997–1.081	0.073
PaCO_2_ (mmHg)	1.015	0.972–1.060	0.500
Left rScO_2_ baseline (%)	1.047	0.899–1.221	0.554
Right rScO_2_ baseline (%)	0.918	0.812–1.039	0.178
Left rScO_2_ mean (%)	1.133	1.001–1.282	0.047 ^*^
Right rScO_2_ mean (%)	1.158	1.013–1.323	0.031 ^*^
Left rScO_2_ minimum (%)	0.993	0.892–1.103	0.898
Right rScO_2_ minimum (%)	1.066	0.955–1.189	0.255
Left rScO_2_ < 55% duration (min)	1.020 1.019(1.005–1.033)	1.005–1.035	0.007 ^*^ 0.007
Right rScO_2_ < 55% duration (min)	1.019	1.005–1.033	0.007 ^*^
Left rScO_2_ < 50% duration (min)	0.991	0.957–1.027	0.628
Right rScO_2_ < 50% duration (min)	1.002	0.990–1.014	0.770
**Multivariable analysis**			
Left rScO_2_ mean (%)	1.098	0.948–1.272	0.212
Left rScO_2_ < 55% duration (min)	1.016	1.002–1.031	0.029 ^*^

Abbreviations: BMI, body mass index; CPB, cardiopulmonary bypass; ACP, antegrade cerebral perfusion

*P < 0.05

**Fig. 3:**
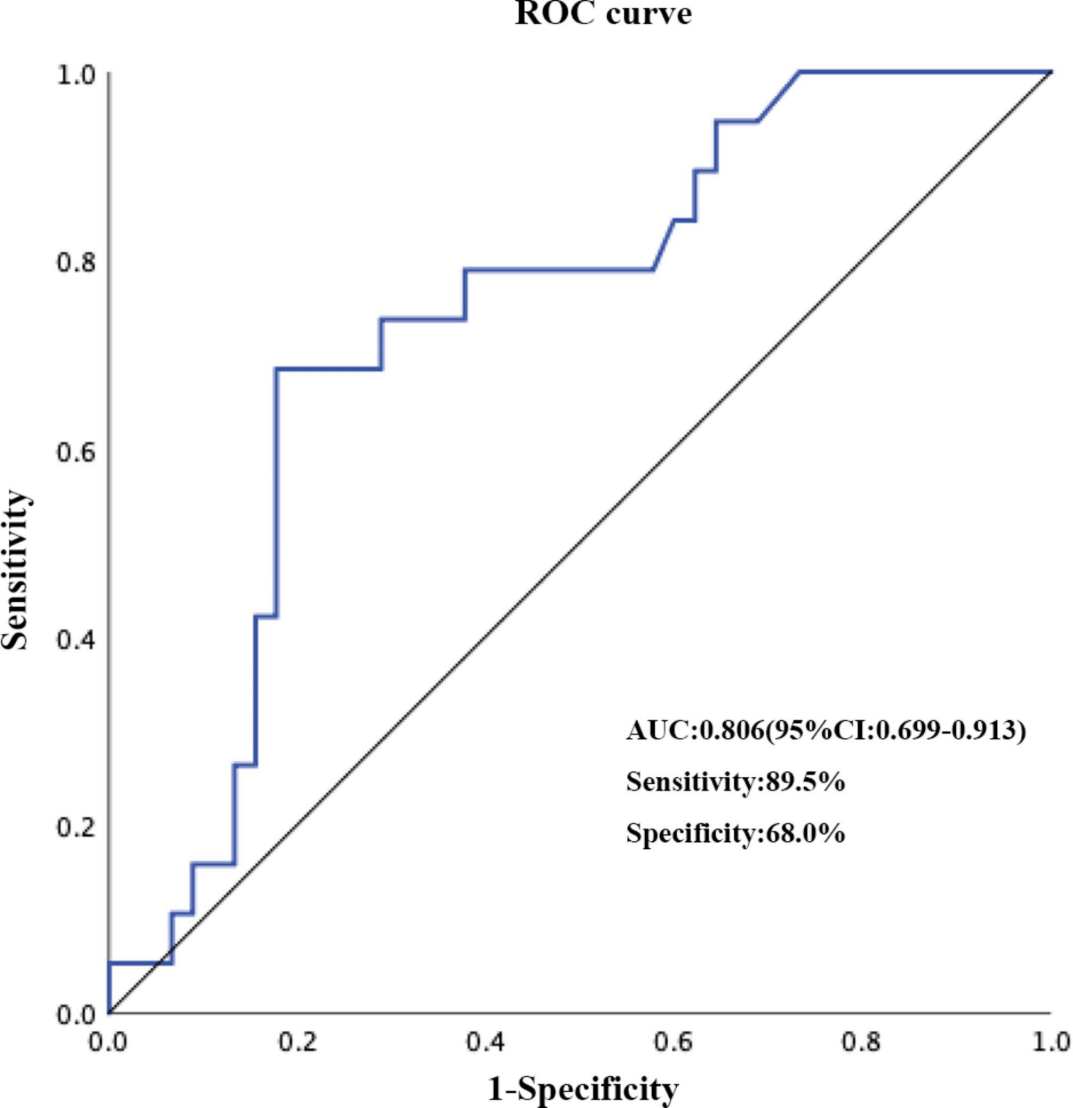
Receiver operating characteristic curve for the duration of low rScO_2_ (rScO_2_ < 55%) identified as independently associated with impaired cerebral autoregulation. AUC, area under the curve; CI, confidence interval

To explore the capacity of rScO_2_ < 55% duration in predicting impaired CA, a receiver operating characteristic curve was applied. The duration of intraoperative rScO_2_ < 55% had an area under the curve of 0.81, with a cutoff value at 40 min (sensitivity, 89.5%; specificity, 68.0%) (Fig. 3).

In-hospital mortality in patients with impaired CA was 26.1% (12/45), of which 33% was cardiogenic shock, 25% neurological, 17% bleeding and 25% other. Compared to patients with normal CA, those who developed impaired CA had higher frequencies of in-hospital mortality, postoperative delirium, AKI, mechanical ventilation > 24 h, and respiratory infection, and prolonged ICU stay. Postoperative cerebral CT was only available in 41 patients with impaired CA and 22 patients with normal CA, with no significant difference in the occurrence of stroke between the two cohorts. There was no significant difference in hospital stay between the 2 cohort (Table 4).

**Table 4:** Outcomes of patients after cardiopulmonary bypass with hypothermic circulatory arrest, stratified by impaired or normal cerebral autoregulation

Outcome	Impaired autoregulation (n = 46)	Normal autoregulation (n = 108)	*P* value
Length of ICU stay (d)	7.3 ± 7.2	4.7 ± 3.5	0.004 ^*^
Hospitalization (d)	19.0 ± 11.1	16.7 ± 7.6	0.146
Re-operation, n (%)	5(10.9)	8 (7.4)	0.946
Ejection fraction < 50%, n (%)	13(28.3)	24(22.2)	0.422
Acute kidney injury, n (%)	17 (37.0)	13(12.1)	< 0.001 ^*^
Delirium, n (%)	31 (67.3)	21(19.4)	< 0.001 ^*^
Postoperative stroke			
Ischemic, n (%)	9 (21.9)	5 (22.7)	0.944
Hemorrhage, n (%)	1 (2.4)	0 (0.0)	0.460
Mechanical ventilation > 24 h, n (%)	32(69.6)	54(50.0)	0.037 ^*^
Lung infection, n (%)	26 (56.5)	39 (36.1)	0.019 ^*^
In-hospital death, n (%)	12(26.1)	9(8.3)	< 0.001 ^*^

Values are n (%) or mean ± SD, unless otherwise noted

Abbreviations: d, days; ICU, intensive care unit

*P < 0.05

## Discussion

In our study, 29.9% of aortic arch surgical patients developed new-onset impaired CA after MHCA and with a worse outcomes. The occurrence of impaired CA in adult patients undergoing MHCA was consistent with children in previous reports [24, 25]. Impairment of CA was more likely to be associated with a prolonged low rScO_2_ (rScO_2_ < 55%), in which the critical threshold of rScO_2_ < 55% duration was 40 min.

It is known that the mechanisms for impaired CA have not yet been elucidated. Notably, there was no association between age, body mass index, gender, diabetes, hypertension, preoperative hemoglobin level and impaired CA. During CPB particularly during HCA and selective cerebral perfusion, factors might influence CA include temperature, PaO_2_, PaCO_2_, perfusion pressure, flow rate, and hematocrit [24–27]. Temperature reduction exponentially decreases cerebral metabolism and preserves cellular stores of high-energy adenosine triphosphate [25]. Carbon dioxide is a potent cerebrovasodilator, and elevated PaCO_2_ can obviously increase CBF volume in both awake and anesthetized states [26]. In our cohort, the patients with impaired or normal CA did not differ significantly in the above factors (Table 2). High PaCO_2_ might be detrimental to preserve the function of CA. And this variable was independently asscociated with impaired CA [1]. In our study, the PaCO_2_ was higher than normal range. However, there was no significant difference between patients with impaired and normal CA. The high PaCO_2_ might be related to that we used pH-stat for blood gas management to ensure sufficient cerebral perfusion during MHCA. Although the seletive cerebral perfusion time showed an obviously difference between impaired CA and normal patients in our study, this variable did not reach a significant association with impaired CA consistent with the result in a literature [20].

Preoperative end-organ malperfusion is common in patients with acute type A aortic dissection, and is associated with significant mortality and morbidity [28]. In particular cerebral malperfusion may result in impairment of the CA. In our study, there was no significant difference in the baseline value of rScO_2_, which could reflect preoperative cerebral perfusion, between patients with impaired CA and normal CA. Other preoperative variables, that may partially reflect organ malperfusion, such as the tamponade, low cardiac output (EF < 50%), creatinine, BNP, and MAP, did not show significant differences between the two cohorts. Nonetheless, we were still unable to assess potential bias in the occurrence of impaired CA because we cannot obtain comprehensive and accurate assessment data on preoperative organ malperfusion by retrospectively reviewing medical records.

We found that impaired CA seems to associate with intraoperative low rScO_2_. The period of rScO_2_ < 55% in impaired CA patients was longer than in normal CA patients. In addition, intraoperative rScO_2_ less than 55% for more than 40 min was independently associated with the onset of impaired CA. This result was consistent with previous studies that the period of rScO_2_ less than 55% during aortic surgery was closely related to the occurrence of postoperative neurological events [20, 21]. These results suggest that regulation cerebral perfusion blood flow rate or pressure alone is not sufficient to prevent the occurrence of rScO_2_ less than 55%. Other methods should also be considered, including switching from unilateral to bilateral cerebral perfusion, increasing hematocrit to improve oxygen delivery, maintaining deep hypothermia to suppress cerebral metabolism, minimizing the duration of HCA, or aggressive upfront antiedema measures [28]. Whereas using α-stat management during moderate hypothermia produces better neurologic outcomes than observed with pH-stat management, it is unclear which strategy is superior in adults when MHCA is used [29].

Our results suggested that patients with impaired CA had a higher rate of postoperative delirium, consistent with previous studies in coronary artery bypass grafting or valve surgery under CPB [30, 31]. Patients with impaired CA were also at increased risks of in-hospital mortality, AKI, mechanical ventilation > 24 h, respiratory infection, and length of ICU stay. Like the present study, other work reported that impaired CA was associated with longer mechanical ventilation and hospital stay [30]. Although there was no significant difference in postoperative stroke between the two cohorts which is inconsistent with the study by Ono M et al., this may be due to the fact that postoperative cerebral CT scan was not performed in all patients in our study [1]. The onsets of AKI, respiratory infection, and postoperative death were affected by many factors, including the cardiac function, bleeding, and the duration of mechnical ventilation. Although the events of low cardiac output and reoperation due to bleeding showed no significant difference between patients with impaired CA and those with normal CA, the causal relationship between impaired CA and postoperative death, AKI and respiratory infection was uncertain from our study which merits prospective studies. Our findings might indicate that impaired CA was one of the manifestations of systemic organ injury in patients who underwent MHCA. These observations suggested the need to comprehensively monitor patients who undergo MHCA to ensure sufficient oxygen delivery to key organs. In particular, patients with impaired CA may require early interventions before postoperative complications onset, such as increasing systemic oxygen delivery, providing renal replacement therapy, and/or giving mild hypothermia therapy.

Our study presents several limitations. First, we were able to enroll only 154 cases because of the relatively small number of total aortic arch replacement surgeries for acute type A aortic dissection at our institution. Second, COx > 0.3 was tested in the animal study as a threshold of impaired CA. Thus, perspective studies were ongoing to explore an absolute value or a certain percentage increase of COx as a measurement tool for impaired CA in adult patients. Third, because rScO_2_ monitoring was not routinely performed after surgery in our center, we could not further calculate postoperative COx to track the duration of impaired CA. Fourth, not all patients received a rigorous assessment by a neurologist or psychiatrist to identify the postoperative neurological complications. This may lead to an underestimation of the occurrence of postoperative neurological complications. In addition, only the temporary rather than permanent neurological complications were evaluated. Fifth, we did not analyze the potential impact of vasoconstrictors or inotropics on CA because the accuracy of the dosage and usage time could not be ensured. Finally, COx was not calculated during CPB, since considering that the site of blood pressure measurement was not constant due to the interruption of perfusion of the aortic arch branches. Furthermore, there is no control group without MHCA in our study. However, the occurrence of new-onset impaired CA in patients undergoing MHCA was higher than that reported in the literature in patients undergoing CPB alone. This might reveal that MHCA increased the risk of new-onset impaired CA.

## Conclusions

Our single-center retrospective study showed that prolonged low rScO_2_ (rScO_2_ < 55%) during aortic arch surgery for type A aortic dissection was closely associated with the development of impaired CA. Impaired CA may be associated with increased rates of postoperative complications and in-hospital mortality.

## Data Availability

The datasets used and analysed during this study are available from the corresponding author on reasonable request.

## List of abbreviations

CA: cerebral autoregulation
CBV: cerebral blood volume
CPB: cardiopulmonary bypass
COx: cerebral oximetry index
HCA: hypothermic circulatory arrest
MHCA: moderate hypothermic circulatory arrest
rScO_2_: regional cerebral oxygen saturation
MAP: mean blood pressure
Mx: mean velocity index
TCD: transcranial Doppler
ECG: electrocardiography
SpO_2_: pulse oxygen saturation
EtCO_2_: end-tidal carbon dioxide
ICU: intensive care unit
CT: computed tomography
AKI: acute kidney injury
KDIGO: Kidney Disease Improving Global Outcomes
EF: ejection fraction
SD: standard deviation
PaCO_2_: carbon dioxide partial pressure
PaO_2_: arterial oxygen partial pressure
BMI: body mass index
ACEI: angiotensin-converting enzyme inhibitor
CCB: calcium-channel blocker
BNP: b-type natriuretic peptide
CVP: central venous pressure
RBC: red blood cell
ACP: antegrade cerebral perfusion
T: temperature
BE: base excess
d: days
